# Dietary supplementation of methionine, lysine, and tryptophan as possible modulators of growth, immune response, and disease resistance in striped catfish (*Pangasius hypophthalmus*)

**DOI:** 10.1371/journal.pone.0301205

**Published:** 2024-04-16

**Authors:** Razia Liaqat, Shafaq Fatima, Wajeeha Komal, Qandeel Minahal, Aya S. Hussain

**Affiliations:** 1 Department of Zoology, Lahore College for Women University, Lahore, Punjab, Pakistan; 2 Department of Biological Sciences, Purdue University Fort Wayne, Fort Wayne, Indiana, United States of America; 3 Department of Forestry and Natural Resources, Purdue University, West Lafayette, Indiana, United States of America; 4 Zoology Department, Faculty of Science, Suez University, Suez, Egypt; Tamil Nadu Dr J Jayalalithaa Fisheries University, INDIA

## Abstract

The present study investigated the potential role of different essential amino acids (AA) in striped catfish (*Pangasius hypophthalmus*). Fish (initial weight = 17.91±0.27 g, n = 260) were fed with eight isonitrogenous (30%), and isolipidic diets (6%) formulated to include different combinations of tryptophan (Trp), methionine (Met), and lysine (Lys) (T0: Zero AA, T1: Trp, T2: Lys, T3: Met, T4: Trp+Met, T5: Lys+Trp, T6: Met+Lys, T7: Lys+Trp+Met) for eight weeks. The dose of amino acid supplementation, whether individually or in combination, was 5g of each amino acid per kg of diet. The trial comprised eight treatments, with each treatment consisted of three replicates (n = 10/replicate). At the end of the growth experiment, the highest total body weight, crude protein, digestive enzymatic activity, immune response, and amino acids level were observed in treatments supplemented with amino acids compared to T0. After the growth experiment, fish in all treatments were exposed to *Staphylococcus aureus* (5×10^5^ CFU/ml). For bacterial challenge trial, the T0 treatment was designated as positive (+ve T0) and negative control (-ve T0). Following the *S*. *aureus* challenge, fish fed with amino acids showed a better response to reactive oxygen species and lipid peroxidation, as indicated by the increased levels of catalase and superoxide dismutase. Conversely, the concentration of malondialdehyde gradually decreased in all treatments compared to the +ve T0 treatment. It is concluded that supplementation of amino acids improved the growth, protein content, and immunocompetency against *S*. *aureus* in striped catfish. The most favorable outcomes in striped catfish were shown by fish supplemented with T7 diet. These essential amino acids hold potential as efficient supplements for use in the intensive aquaculture for striped catfish.

## 1. Introduction

Striped catfish (*Pangasius hypophthalmus*), is an economically valuable fish and currently ranks among the most traded freshwater fish species worldwide [1]. The global production of this species was recorded as 2520.41 thousand tons in 2022 [2]. Enhancing catfish production can be achieved by emphasizing on high-quality feed [3], particularly by aligning the nutritional content of feed with specific dietary requirements [4, 5]. The demand for fishmeal and fish oil as feed components has increased dramatically to meet the rising needs of aquaculture production [6]. However, due to higher prices and limited supply of fishmeal, it has become necessary to search for alternative plant protein sources. It is important to consider that such alternative sources should have balanced dietary components and be capable of maintaining high growth rates [7]. High-quality feed is not limited to protein content alone but also encompasses essential amino acids to promote growth performance [8]. These amino acids greatly influence the growth patterns, reproductive performance, and development of a fish [9].

Among these essential amino acids, lysine is one of the most limiting amino acids in fish feed [10]. Lysine is found in significant proportions in fish muscle tissues such as rohu (*Labeo rohita*) (2.9%) [6], catla (*Catla catla*) (3.6g%) [11], juvenile black sea bream (*Sparus aurata*) (6.63%) [12], basa (*Pangasius bocourti*) (8.41%) [13]. It plays a pivotal role in growth [14], maintaining nitrogen balance [15], preventing excessive fat accumulation in the body [16], and maintaining osmotic pressure and acid-base balance in the body fluids [17]. It also known for inhibiting fin rotting of a fish, decreasing the mortality rates [18], enhancing protein deposition in the body and fillet content [19–21], increasing muscle growth in fish by rapidly increasing the size and length of muscle fibers through hyperplasia and hypertrophy [22], and being involved in the facilitation of ‘cross-linking’ protein, especially collagen [23]. The required range of lysine as a fish feed component varies between 2 to 4% of the total dietary protein for different fishes [24]. Furthermore, all finfish species require lysine as an essential dietary component, especially when alternative protein sources are used instead of fishmeal. In addition, fish fed with diets deficient in this essential amino acid showed reduced growth, and higher mortality rates [25].

After lysine, methionine is the second limiting amino acid in plant protein sources [5, 26]. These amino acids are involved in the synthesis of carnitine, which functions in the transportation of fatty acids to produce energy through oxidation [27], and increase protein retention [28]. Methionine supplementation can enhance protein synthesis in fish, as seen in blackhead seabream (17.25%) [29], improve growth performance in several fish species including common carp (*Cyprinus carpio*) [30], channel catfish (*Ictalurus punctatus*) [31], northern snakehead (*Channa argus*) [32], nile tilapia (*Oreochromis niloticus*) [33]. It also helps in maintaining antioxidant system [34], and enhance energy metabolism by synthesizing cysteine and glutathione through trans-sulphuration pathway [35]. On the other hand, studies have indicated that methionine deficiency leads to decreased survival rates, and reduced growth performance in Jian carp (*Cyprinus carpio*) [36], rainbow trout (*Oncorhynchus mykiss*) [37], juvenile red drum (*Sciaenops ocellatus*) [38]. Additionally, methionine deficiency can lead to the development of lenticular cataracts in seabream [39].

In contrast to methionine and lysine, tryptophan serves as the precursor for both serotonin (5-hydroxytryptamine, 5-HT) and melatonin (N-acetyl-5- methoxytryptamine). These compounds have significance in fortifying the immune response [40–42], improve growth performance [43], exert significant immunomodulatory influence, and function as potent scavengers of deleterious free radicals [44]. Numerous independent investigations have explored the effect of tryptophan, lysine, and methionine on growth performance and immune response of various fish species [45–50], including striped catfish [51], basa catfish [52]. To the best of our knowledge, there is no existing published data available on synergistic impact of these three amino acids on growth performance, disease resistance, and immunocompetency of striped catfish. Therefore, the purpose of the present investigation was to assess the potential influence of these essential amino acids on the growth, protein content, digestive enzymes, and immune response in striped catfish. The concentrations of these amino acids were selected based on the dietary requirement of catfish.

## 2. Materials and methods

### 2.1 Preparation of experimental diets

Isonitrogenous (30% crude protein) and isolipidic (6%) diets were formulated. Treatment diets were prepared by mixing the finely ground ingredients (grains were procured from local farmers in Pakistan while origin of soybean was USA; Table 1) with different combinations of methionine (Met), lysine (Lys), and tryptophan (Trp) (T0; zero supplementation, T1; Trp, T2; Lys; T3; Met: T4; Trp + Met, T5; Lys +Trp, T6; Met +Lys, T7; Lys + Trp +Met). L- lysine, L- methionine, and L- tryptophan were purchased (Sigma Aldrich, USA). A total of 0.50% of each amino acid was added to one kg of feed in each diet according to catfish dietary requirement [50]. All ingredients were thoroughly mixed and pellets (1mm) were made by using mechanical pellet machine (manufactured by PCSIR laboratories, Pakistan). The pellets were air-dried at room temperature and stored at 4°C. Ingredients and chemical composition of the treatment diets are given in Table 1. Amino acids profile of treatment diets is given in Table 2. Chemical composition and amino acid analysis of treatment diets were determined as mentioned in section 2.4.

**Table 1:** Ingredients and chemical composition of experimental diets.

Ingredients (%)	T0	T1	T2	T3	T4	T5	T6	T7
Corn meal	28.00	28.00	28.00	28.00	28.00	28.00	28.00	28.00
Rice polish	13.00	13.00	13.00	13.00	13.00	13.00	13.00	13.00
Wheat bran	8.00	8.00	8.00	8.00	8.00	8.00	8.00	8.00
Canola meal	7.00	7.00	7.00	7.00	7.00	7.00	7.00	7.00
Soybean meal	39.00	39.00	39.00	39.00	39.00	39.00	39.00	39.00
Dicalcium phosphate	3.00	3.00	3.00	3.00	3.00	3.00	3.00	3.00
Methionine	0.19	0.19	0.19	0.19	0.19	0.19	0.19	0.19
Lysine	1.58	1.58	1.58	1.58	1.58	1.58	1.58	1.58
L-Threonine	0.39	0.39	0.39	0.39	0.39	0.39	0.39	0.39
Trp (5g/Kg)	0.00	0.50	0.00	0.00	0.00	0.00	0.00	0.00
Lys (5g/Kg)	0.00	0.00	0.50		0.00	0.00	0.00	0.00
Met (5g/Kg)	0.00	0.00	0.00	0.50	0.00	0.00	0.00	0.00
Trp + Met (5g/Kg)	0.00	0.00	0.00	0.00	0.50+0.50	0.00	0.00	0.00
Lys + Trp (5g/Kg)	0.00	0.00	0.00	0.00	0.00	0.50+0.50	0.00	0.00
Met + Lys (5g/Kg)	0.00	0.00	0.00	0.00	0.00	0.00	0.50+0.50	0.00
Lys +Trp + Met (5g/Kg)	0.00	0.00	0.00	0.00	0.00	0.00	0.00	0.50+0.50+0.50
**Chemical Composition of Feed**								
Moisture (%)	10.02	10.12	10.20	10.03	10.21	10.01	10.02	10.13
Crude Protein (%)	29.99	30.10	30.10	30.03	30.12	30.11	30.09	30.12
Crude Fat (%)	6.16	6.16	6.18	6.19	6.17	6.19	6.18	6.17
Crude Ash (%)	8.72	8.81	8.61	8.90	8.93	8.82	8.74	8.76

(T0; C, T1; Tryptophan, T2; Lysine; T3; Methionine: T4; Tryptophan + Methionine, T5; Lysine +Tryptophan, T6; Methionine +Lysine, T7; Lysine + Tryptophan +Methionine). Tryptophan: (Trp), Methionine: (Met), Lysine: (Lys)

**Table 2:** Profile of amino acids (% of complete diet) in experimental diets.

Amino Acids	T0	T1	T2	T3	T4	T5	T6	T7
Methionine	0.11±0.02	0.19±0.12	0.21±0.02	0.65±0.22	0.65±0.01	0.11±0.21	0.65±0.22	0.65±0.02
Tryptophan	0.11±0.01	0.61±0.20	0.11±0.01	0.11±0.02	0.59±0.13	0.59±0.01	0.11±0.03	0.59±0.01
Valine	7.23±0.02	6.90±0.11	7.12±0.03	7.30±0.02	7.40±0.01	7.31±0.05	7.23±0.02	7.23±0.04
Isoleucine	1.80±0.04	1.81±0.21	1.81±0.12	1.80±0.22	1.82±0.12	1.81±0.02	1.83±0.14	1.82±0.15
Leucine	0.46±0.21	0.40±0.01	0.41±0.21	0.41±0.01	0.41±0.20	0.42±0.20	0.41±0.04	0.41±0.01
Phenylalanine	5.40±0.04	5.32±0.21	5.40±0.16	5.43±0.12	5.43±0.14	4.43±0.21	0.53±0.01	5.43±0.02
Histidine	0.07±0.04	0.07±0.01	0.07±0.03	0.08±0.13	0.08±0.12	0.08±0.10	0.08±0.04	0.09±0.01
Lysine	1.50±0.02	1.50±0.02	2.40±0.02	1.51±0.12	1.51±0.04	2.41±0.02	2.42±0.02	2.43±0.10
Ornithine	3.33±0.21	3.32±0.12	3.34±0.03	3.34±0.14	3.21±0.06	3.33±0.04	3.40±0.11	3.22±0.02
Cysteine	0.02±0.11	0.03±0.14	0.02±0.13	0.03±0.20	0.03±0.14	0.03±0.15	0.03±0.13	0.09±0.11
Aspartic Acid	0.22±0.04	0.23±0.22	0.22±0.02	0.23±0.14	0.23±0.01	0.24±0.02	0.23±0.04	0.23±0.02
Asparagine	1.55±0.22	1.56±0.32	1.56±0.03	1.57±0.15	1.57±0.03	1.58±0.02	1.58±0.03	1.53±0.01
Serine	0.21±0.01	0.21±0.12	0.21±0.13	0.21±0.05	0.22±0.13	0.23±0.15	0.21±0.16	0.22±0.17
Glycine	0.005±0.23	0.006±0.02	0.005±0.03	0.007±0.16	0.008±0.03	0.007±0.05	0.006±0.01	0.008±0.01
Alanine	1.36±0.11	1.36±0.02	1.36±0.13	1.37±0.14	1.35±0.13	1.36±0.11	1.37±0.11	1.36±0.13
Tyrosine	0.04±0.01	0.05±0.12	0.05±0.03	0.05±0.05	0.06±0.04	0.04±0.02	0.05±0.05	0.05±0.02
Threonine	6.08±0.21	6.11±0.04	5.91±0.02	6.03±0.14	5.83±0.13	6.11±0.05	6.02±0.04	6.11±0.02

(T0; C, T1; Tryptophan, T2; Lysine; T3; Methionine: T4; Tryptophan + Methionine, T5; Lysine +Tryptophan, T6; Methionine +Lysine, T7; Lysine + Tryptophan +Methionine), Tryptophan (Trp), Methionine (Met), Lysine (Lys).

### 2.2 Growth experiment

Trial was started after ethical approval from animal ethics committee (Ref. No.: Zoo/LCWU/932). Fish were collected from a local hatchery (Lahore, Pakistan) and transported to the aquaculture facility at Lahore College for Women University. Fish were acclimatized in three 600L tanks for a week and fed with prepared feed without any supplementation (diet in T0). After acclimatization, fish (initial weight = 17.91±0.27g, total no. = 260) were stocked in 24 (150L) glass aquaria (stocking density: 1.19kg/m^3^) for eight weeks. Each of the eight treatments had three replicates, while each replicate had ten fish. Remaining twenty fish were fed with a diet without amino acids supplementation to be used as the negative control in the bacterial challenge trial (section 2.3). These fish were reared in two separate glass aquaria, (10 fish each) under the same husbandry conditions. Daily ration was calculated based upon 2% of the biomass weight and each treatment was fed three times a day. A total of 10% water was exchanged on daily basis to maintain water quality at adequate level for the fish. Fish were reared at ambient temperature and photoperiod. The water quality parameters including dissolved oxygen (DO) (7.51±0.21mg/L), pH (7.21±0.41), and water temperature (29.00±1.00°C) were monitored on a daily basis. Ammonia, nitrite and nitrate in water were tested once a week and their values were noted to be lower than detection limit of water quality testing kits (API freshwater test kit, USA; LE144RS). Fish behavior was monitored on daily basis.

### 2.3 Bacterial challenge

#### 2.3.1 Isolation of *Staphylococcus aureus*

*S*. *aureus* was obtained from diseased *Labeo rohita* fish originating from the University diagnostic laboratory, Department of Microbiology, University of Veterinary and Animal Sciences, Lahore Pakistan. A 10-gram portion of the diseased fish muscle sample was blended with 90 ml of sterile peptone water, generating a 1:10 dilution, to facilitate the enrichment of the target bacterial species. Subsequently, this mixture was incubated at 37°C for 6 hours following Akbar and Anal [53]. From dilutions, 0.5 ml was inoculated on to Mannitol Salt Agar (MSA) and incubated at 37°C for 24 hours. The emergence of colonies exhibiting a yellow hue was indicative of *S*. *aureus* and was subsequently validated through gram staining and coagulase production test using a Pastorex Staph Plus kit (Bio-Rad, Hercules, CA). The purified subculture was duly preserved to facilitate subsequent analyses.

#### 2.3.2 Challenge with *S*. *aureus*

After the growth experiment, fish were challenged with *S*. *aureus* for 15 days. The *S*. *aureus* culture was prepared in 10 ml volume of nutrient broth (HiMedia Ltd., Lahore, Pakistan). Subsequently, the culture was vortexed, and incubated in shaker incubator for 24 hours at 37°C. The culture was centrifuged (Micro Prime Centrifuge, Pocklington, UK) at 5000 rpm for 15min at 4°C to get the hard pellet at the bottom. The obtained pellet underwent several washings, employing sterile phosphate buffer saline (PBS). Following thorough washing, the pellet was re-suspended in PBS (pH 7.4). To ascertain the optical density of bacterial suspension, a UV spectrophotometer was utilized to obtain concentration of 5×10^5^ CFU/ml. The control group was split into two distinct subgroups: positive control (+ve T0) and negative control (-ve T0). Fish in -ve T0 was given bath with PBS only, whereas the other groups (+ve T0, T1, T2, T3, T4, T5, T6, and T7) (n = 15 for each treatment) were exposed to *S*. *aureus* (5×10^5^ CFU/ml). Fish were bathed for 2 hours and the bath was repeated after seven days. Throughout the challenge period, all fish in all treatments were fed with their relevant diets.

### 2.4 Sample collection

At the end of the experiment, fish were fasted for 24 hours and anesthetized using clove oil (6ml/L; Sigma Aldrich USA) for five minutes. Five fish were randomly collected from each replicate of each treatment after growth experiment. All remaining fish were sampled after bacterial challenge. Total body weight, total body length, specific growth rate (SGR), feed conversion ratio (FCR), condition factor, and weight of viscera, and liver were calculated by using following formulae:

$$Specific\;growth\;rate\;\left(\frac{\%}{day}\right)=\frac{\left[(\ln\;final\;weight-\ln\;Initial\;weight)\right]}{\left[Final\;time–Initial\;time\right]}\times 100$$

$$Condition\;factor\;(K)=\left[\frac{Weight\;}{{Length}^{3}\;}\right]\times 100$$

$$Viscerosomatic\;index\;(VSI)\;(\%)=\left[\frac{Visceral\;weight\;}{Total\;body\;weight}\right]\times 100$$

$$Hepatosomatic\;index\;(HSI)\;(\%)=\left[\frac{Weight\;of\;liver\;}{Total\;body\;weight}\right]\times 100$$

$$Feed\;conversion\;ratio\;(FCR)=\frac{Total\;feed\;given\;(Dry\;weight)}{Total\;weight\;gain\;(Wet\;weight)}$$

$$Survival\;rate\;(\%)=\left[\frac{Number\;of\;surviving\;fish}{Initial\;number\;of\;fish}\right]\times 100$$

Blood was collected from caudal vein and stored in pro-coagulation clot activator (Xiangyuan Medical, Germany) and EDTA coated tubes (Xinle, China), respectively. Clot activator tubes were used to collect serum, while EDTA coated tubes were utilized for analysis of hematology and blood biochemistry. Blood samples were centrifuged at 5000 rpm for 20 min to extract plasma. It was stored at -20°C until assessed. Muscle samples were collected and stored at -20°C to determine chemical composition, and amino acid profile. Whereas, intestine samples were collected, washed with autoclaved sterile water, centrifuged at 5000 rpm, pellet collected at the bottom of centrifuge tubes and stored at 4°C to determine digestive enzymes. Fish muscles, liver, kidney, gut, and gills were collected for histological analysis.

### 2.5 Chemical composition and amino acid analysis

The chemical composition of muscles was analyzed using the protocol outlined by the association of official analytical chemists [54]. Muscle samples were dried in an oven at 80°C until a constant dry weight was achieved. The dried samples were then ground for further chemical analysis. Crude protein was determined using the Kjeldahl apparatus (PCSIR Laboratories, Pakistan). Crude lipids were determined following Folch method [55] in the Soxhlet apparatus (PCSIR Laboratories, Pakistan). The ash content in the muscles were determined by using furnace (PCSIR Laboratories, Pakistan). An amino acid analyzer (Biochrome 30+, Biochrome limited, Cambridge, UK) was used to quantify the amino acid contents of fish muscles and the analytical protocols followed by Ahmad et al. [56].

### 2.6 Digestive enzymes assay

Protease, amylase, and lipase enzymatic extracts from intestine samples were prepared following a method by Ding et al. [57]. Intestine samples were rinsed with sterile water and homogenized in phosphate buffer saline (PBS, 1g/10ml; pH = 7.5), and centrifuged at 5000 rpm for 20 minutes. The supernatant was collected and preserved at 4°C. Protease activity of intestine samples was determined using Folin-phenol reagent, according to Jin [58]. Quantification of amylase enzymes activity was carried out by utilizing iodine to detect the unhydrolyzed starch in samples, followed by Jiang [59]. Lipase activity was assessed by measuring the fatty acids released through the enzymatic breakdown of triglycerides, as described by Borlongan [60]. The enzymatic activities are expressed as intestine content units per liter (U/L).

### 2.7 Hematology, blood biochemistry and assays of antioxidant biomarkers

The levels of haemoglobin (g/dl), white blood cells (μL), red blood cells (μL), mean corpuscle volume (MCV; fL), haematocrit (HCT; %), platelets (μL), mean corpuscular haemoglobin (MCH; %), neutrophils (μL), lymphocytes (μL), monocytes (μL), and eosinophils (μL), were measured by using clinical hematology analyzer (Sysmex, China; KX-21N). Blood glucose level (mg/dl) was measured by using laboratory blood glucose analyzer (MegMedius, Karachi Pakistan; GLM-78). Triglycerides (mg/dl) were measured by using ELISA (Biocompare, USA) as per manufacturer’s protocol. Alanine aminotransferase (ALT; Unit/L), and aspartate aminotransferase (AST; Unit/L) were analyzed using analytical kits (Thermo Fisher Scientific, USA; Catalog # A7528-150, Catalog# A7561-150) on a clinical chemistry analyzer (Thermo Fisher Scientific, USA).

The SOD activity [enzyme commission number: EC.1.15.1.1], was assessed utilizing the SOD ELISA Kit (Pars Biochem; catalog# PRS-02005 hu). SOD activity was determined by measuring the auto-oxidation rates in the presence and absence of the sample, and results were expressed as μmol/L. The activity of catalase (enzyme commission number: EC.1.11.1.6) were determined spectrophotometrically (560nm) using catalase colorimetric activity kit (Thermo fisher scientific, USA; catalog# EIACATC), as per manufacturer instruction. Malondialdehyde (MDA; enzyme commission number: EC. 202-974-4) concentration was determined using ELISA Kit (catalog# PRS - 00991hu). MDA level was measured within the range of 0.3nmol/ml- 7nmol/ml at 450nm.

### 2.8 Histological analysis

At the end of the bacterial challenge, the intestine, gills, liver, muscles, and kidney were collected from each treatment (n = 5 of each organ), and placed in sterilized tubes containing 3ml of Bouin’s solution (Solarbio, Beijing, China). Following the samples were dehydrated and embedded in paraffin. Sections with a thickness of 5μm were then sliced from each sample and subjected to staining with hematoxylin and eosin according to Humason, [61].

### 2.9 Statistical analysis

Results were presented as mean ± standard error (S.E). Statistical analysis of the data was performed using one-way analysis of variance (ANOVA) with a significance level set at *P*< 0.05 to determine significant differences among groups. Based on the normality (Kolmogorov–Smirnov test) and Levene test of homogeneity of variances, variance between means were further analyzed using Duncan Multiple Range Test (DMRT). The parameters which showed significant variance after DMRT test have been mentioned with superscripts. All the Analyses were performed using SPSS version 20 (Armonk, NY: IBM Corp).

## 3. Results

### 3.1 Growth

A significant difference (*P*<0.05) was observed in all growth parameters among the eight treatments (Table 3). These parameters gradually increased in treatments with combined amino acids, compared to treatments supplemented with individual amino acids. All treatments showed a significant increase in all growth parameters compared to the T0 treatment, except for SGR. The SGR between T0, T1, and T2 showed insignificant differences, whereas all other treatments showed significant difference. The T0 treatment showed the lowest total body weight (38.82±0.28 g), and SGR (0.56±0.005%/day). The HSI between T0, T1, T2, and T3 showed an insignificant difference (*P*>0.05), whereas, T4, T5, T6, and T7 showed a significant increase (*P*<0.05). The T7 treatment showed the highest value of total body weight (58.21±1.25 g), SGR (1.07±0.005%/day), K (1.37±0.01%), HSI (2.68±0.09%), and VSI (4.85±0.23%). Similarly, the best FCR (0.71±0.004%) was also recorded in fish fed with T7 treatment.

**Table 3:** Summary of growth parameters in eight treatments at the end of the growth experiment. Different superscripts across the rows represent the variance between treatments were calculated by Duncan multirange test of one-way ANOVA at *P* < 0.05.

Parameters	T0	T1	T2	T3	T4	T5	T6	T7
TBW (g)	38.82±0.28^a^	39.52±0.36^b^	38.51±0.42^c^	38.82±0.27^d^	45.27±0.31^e^	43.58±0.68^f^	45.56±0.31^g^	58.21±1.25^h^
TBL (cm)	12.00±0.12^a^	12.78±0.42^c^	12.08±0.27^b^	12.00±0.22^a^	14.31±0.28^d^	14.54±0.21^e^	14.31±0.33^d^	16.21±0.41^f^
SGR (%/day)	0.56±0.005^a^	0.56±0.005^a^	0.56±0.005^a^	0.61±0.008^b^	0.78±0.005^d^	0.97±0.005^e^	0.71±0.003^c^	1.07±0.005^f^
K (%)	2.25±0.005^f^	2.25±0.005^f^	2.17±0.008^e^	1.89±0.01^d^	1.55±0.005^c^	1.54±0.005^c^	1.42±0.008^b^	1.37±0.005^a^
HSI (%)	2.05±0.02^a^	2.05±0.06^a^	2.05±0.02^a^	2.05±0.06^a^	2.53±0.06^c^	2.53±0.06^c^	2.41±0.11^b^	2.68±0.09^d^
VSI (%)	2.48±0.04^b^	2.48±0.35^b^	2.48±0.04^b^	2.41±0.06^a^	4.01±0.14^d^	4.01±0.14^d^	3.65±0.07^c^	4.85±0.23^e^
FCR	1.31±0.005^g^	1.31±0.005^g^	1.23±0.008^e^	1.29±0.008^f^	0.99±0.005^c^	1.03±0.005^d^	0.95±0.01^b^	0.71±0.004^a^

T0; control, T1; Tryptophan, T2; Lysine; T3; Methionine: T4; Tryptophan + Methionine, T5; Lysine +Tryptophan, T6; Methionine +Lysine, T7; Lysine + Tryptophan +Methionine). TBW- total body weight, TBL- total body length, SGR- specific growth rate, K- condition factor, HSI-hepatosomatic index, VSI- viscerosomatic index, FCR- feed conversion ratio

### 3.2 Chemical composition and amino acid profile of muscles

Chemical composition (moisture content, crude protein, crude fat and crude ash) showed a significant difference (*P*<0.05) among all treatments at the end of the growth experiment (Table 4). The crude fat was significantly decreased among treatments supplemented with amino acids (*P*<0.05). The highest moisture content was observed in the T0 treatment compared to other treatments. The lowest crude ash was observed in T2 and T7 treatments compared to the other treatments. The crude protein in T0 treatment was the lowest (16.76±0.19), compared with other treatments. The highest concentration of crude protein (22.75±0.01%) was observed in T7 treatment. Results showed a significant difference (*P*<0.05) between essential amino acids (EAA) especially lysine, tryptophan, and methionine mostly among all treatments (Table 5). Treatments without amino acids (T0) had a significantly (*P*<0.05) lower concentration of essential amino acids (EAAs) and non-essential amino acids (NEAA) concentrations compared to other treatments (Table 5). The methionine (0.66±0.04%), lysine (2.46±0.08%), and tryptophan (0.53±0.04%) in the T0 treatment were significantly lower (*P*<0.05) compared to the other treatments. The significantly highest amino acids concentrations, methionine (0.99±0.02%), lysine (4.14±0.72%), and tryptophan (0.98±0.02%) were observed in T7 treatment. The amino acids arginine, alanine, and carnitine showed insignificant (*P*>0.05) differences between the T0, T1, and T2 treatment compared with others (Table 5).

**Table 4:** Chemical composition of muscles in eight treatments at the end of the growth experiment. Different superscripts across the rows represent the variance between treatments were calculated by Duncan multirange test of one-way ANOVA at *P* < 0.05.

Parameters	T0	T1	T2	T3	T4	T5	T6	T7
Moisture (%)	70.21±0.93^g^	68.21±0.57^e^	69.00±0.98^c^	64.32±0.93^b^	67.21±0.57^c^	66.16±0.83^b^	63.01±0.73^a^	69.01±0.98^f^
Crude Protein (%)	16.76±0.19^a^	17.45±0.15^b^	17.45±0.15^b^	18.37±0.03^c^	19.25±0.01^d^	20.12±0.08^e^	21.86±0.02^f^	22.75±0.01^g^
Crude Fat (%)	8.26±0.01^d^	9.45±0.11^f^	8.51±0.14^e^	8.52±0.14^e^	7.47±0.07^b^	7.12±0.34^a^	7.75±0.007^c^	7.75±0.007^c^
Crude Ash (%)	4.43±0.16^c^	4.65±0.02^d^	4.21±0.02^a^	5.57±0.15^f^	5.96±0.01^h^	5.86±0.01^g^	4.92±0.01^e^	4.35±0.02^b^

(T0; control, T1; Tryptophan, T2; Lysine; T3; Methionine: T4; Tryptophan + Methionine, T5; Lysine +Tryptophan, T6; Methionine +Lysine, T7; Lysine + Tryptophan +Methionine).

**Table 5:** Essential amino acids (EAA) and non-essential amino acids (NEAA) contents from fish muscles from eight treatments at the end of the growth experiment; expressed as %. Different superscripts across the rows represent the variance between treatments were calculated by Duncan multirange test of one-way ANOVA at *P* < 0.05.

**EAA**	**T0**	**T1**	**T2**	**T3**	**T4**	**T5**	**T6**	**T7**
Methionine	0.34±0.04^a^	0.42±0.04 ^c^	0.41±0.04^b^	0.97±0.04^f^	0.99±0.02^f^	0.69±0.02^d^	0.78±0.05^e^	0.99±0.02^g^
Tryptophan	0.53±0.04^a^	0.68±0.04^d^	0.58±0.01^b^	0.59±0.02^bc^	0.79± 0.01^f^	0.85± 0.03^g^	0.59±0.01^c^	0.98±0.02^h^
Valine	1.97±1.06^a^	2.20± 0.48^b^	2.36±0.07^c^	2.50±0.11^e^	2.40±0.81^d^	2.71±0.27^e^	2.81±0.69^f^	3.20±0.58^g^
Isoleucine	3.60±0.05^a^	3.93± 0.05^b^	4.92±0.08^c^	5.20±0.67^d^	5.70±0.38^f^	5.90±0.03^g^	5.50±0.01^e^	9.80±3.97^h^
Leucine	0.36±0.05^a^	0.65±0.07^e^	0.57±0.01^d^	0.65±0.06^e^	0.78±0.04^f^	0.42±0.01^b^	0.52±0.02^c^	1.16±0.04^f^
Phenylalanine	1.12±0.04^a^	1.43±0.04^b^	1.65±0.05^c^	1.46±0.03^b^	1.88±0.04^d^	2.26±0.01^f^	2.02±0.05^e^	2.33±0.06^g^
Histidine	0.19±0.05^c^	0.12±0.05^a^	0.13±0.02^b^	0.13±0.01^b^	0.25±0.05^d^	0.26±0.04^e^	0.28±0.04^f^	0.45±0.04^g^
Lysine	2.46±0.08^a^	2.47±0.01^b^	3.48±0.51^d^	2.62±0.75^c^	2.72±0.41^d^	3.74±0.55^e^	3.99±0.02^f^	4.14±0.72^g^
Arginine	0.97±0.04^bc^	0.99±0.01^b^	0.99±0.02^b^	1.03±0.04^c^	1.03±0.04^c^	1.12±0.37^d^	1.16±0.04^e^	1.19±0.04^f^
Ornithine	1.22±0.71^a^	1.32±0.71^b^	2.70±0.58^d^	1.52±0.67^c^	2.80±0.67^e^	3.30±0.97^h^	3.21±0.61^g^	2.92±1.85^f^
TEAA	13.08	14.47	18.05	16.67	19.34	18.25	17.97	27.16
**NEAA**	**T0**	**T1**	**T2**	**T3**	**T4**	**T5**	**T6**	**T7**
Cysteine	0.09±0.01^a^	0.13±0.01^b^	0.17±0.01^d^	0.39±0.03^e^	0.15±0.01^c^	0.17±0.04^d^	0.47±0.03^f^	0.59±0.02^g^
Aspartic Acid	4.60±0.58^a^	4.80±0.05^b^	5.21±0.03^e^	5.01±0.03^d^	5.42±0.02^f^	5.60±0.08^g^	4.90±0.19^c^	5.72±0.04^h^
Asparagine	7.60±0.03^a^	8.90±0.01^c^	9.22±0.07^d^	9.22±0.12^d^	8.77±0.04^b^	9.62±0.04^c^	9.92±0.05^d^	10.02±0.06^e^
Serine	4.23±0.39^a^	4.90±0.59^d^	4.60±0.05^c^	4.42±0.03^b^	5.12±0.04^e^	5.30±0.04^f^	5.82±0.03^g^	6.40±0.02^h^
Glutamine	2.80±0.59^a^	3.10±0.01^b^	2.92±0.59^ab^	4.52±0.81^c^	5.16±0.61^d^	5.60±0.81^de^	6.63±0.58^e^	8.42±0.64^f^
Glycine	5.62±0.01^a^	6.87±0.01^b^	7.33±0.62^d^	7.19±0.64^c^	7.56±0.03^e^	7.35±0.07^d^	7.48±0.04 ^de^	8.62±1.01^f^
Alanine	2.80±0.21^a^	2.85±0.03^a^	2.97±0.03^b^	3.20±0.03^c^	6.30±0.05^e^	6.22±0.05^e^	6.95±0.03^d^	7.80±0.05^f^
Proline	6.92±0.01^a^	7.07±0.04^b^	7.24±0.03^cd^	7.15±0.05^bc^	7.35±0.05^d^	7.41±0.57^d^	7.40±0.58^d^	7.64±0.39^e^
Tyrosine	0.25±0.07^b^	0.30±0.09^c^	0.69±0.03^a^	0.29±0.01^c^	0.35±0.04^d^	0.23±0.01^e^	0.44±0.05^f^	0.57±0.06^g^
Ornithine	1.22±0.71^a^	1.21±0.58^a^	1.62±0.67^b^	1.90±0.67^d^	1.90±0.9^d^	1.60±0.41^b^	1.82±1.85^c^	2.01±0.61^e^
TNEAA	36.13	39.04	41.97	43.29	48.08	49.1	51.83	57.79

(T0; control, T1; Tryptophan, T2; Lysine; T3; Methionine: T4; Tryptophan + Methionine, T5; Lysine +Tryptophan, T6; Methionine +Lysine, T7; Lysine + Tryptophan +Methionine). TEAA: total essential amino acids, TNEAA: total non-essential amino acids

### 3.3 Digestive enzymes assay

Dietary supplementation of amino acids significantly (*P*<0.05) increased the levels of amylase, lipase, and protease in the intestine (Table 6). The T0 treatment showed the lowest protease (35.01±0.57 unit/L), lipase (136.00± 0.57 unit/L), and amylase (20242.01±0.88 unit/L) at the end of the growth experiment. The protease, lipase, and amylase level were significantly (*P*<0.05) higher in T4, T5, and T6 treatments compared to T1, T2, and T3 treatments. The highest level of digestive enzymes was observed in T7 treatment. The T7 treatment showed the highest protease (55.21±0.57 unit/L), lipase (182.12±1.15 unit/L), and amylase (40411.03±0.66 unit/L) at the end of growth experiment.

**Table 6:** Determination of digestive enzymes in fish intestine samples from all eight treatments at the end of the growth experiment. Different superscripts across the rows represent the variance between treatments were calculated by Duncan multirange test of one-way ANOVA at *P* < 0.05.

Parameters	T0	T1	T2	T3	T4	T5	T6	T7
Amylase (Unit/L)	20242.01±0.88^b^	20241.23±0.57^a^	20251.51±0.57^c^	20261.11±0.57^d^	37961.00±0.57^g^	37952±0.57^f^	36972.41±0.57^e^	40411.03±0.66^h^
Lipase (Unit/L)	136.00± 0.57^a^	142.04±1.15^c^	142.05±1.15^c^	142.00±0.88^b^	161.21± 0.57^e^	146.13±1.15^d^	172.12± 0.57^f^	182.12±1.15^g^
Protease (Unit/L)	35.01±0.57^a^	39.34±0.57^c^	45.41±0.57^d^	37.23±0.57^b^	53.03±0.57^g^	49.06±0.57^f^	47.01± 0.57^e^	55.21±0.57^h^

(T0; control, T1; Tryptophan, T2; Lysine; T3; Methionine: T4; Tryptophan + Methionine, T5; Lysine +Tryptophan, T6; Methionine +Lysine, T7; Lysine + Tryptophan +Methionine).

### 3.4 Hematology, blood biochemistry and antioxidant enzymes assay

**Fig 1:**
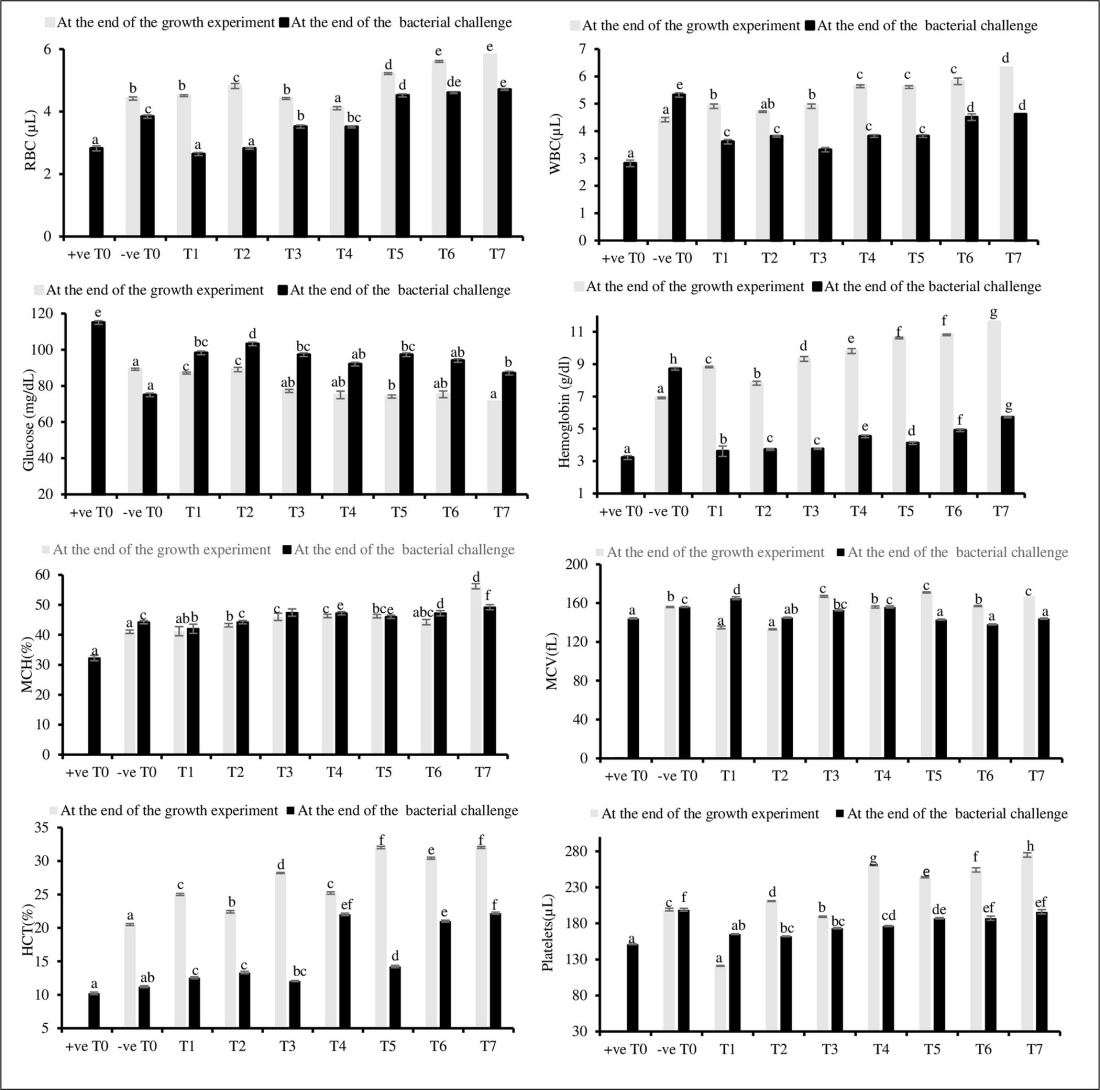
Analysis of hematological parameters in eight treatments at the end of the growth experiment and after bacterial challenge. Different superscripts represent the variance between treatments were calculated by Duncan multirange test of one-way ANOVA at *P* < 0.05. different treatments are as follows: T0; control, T1; Tryptophan, T2; Lysine; T3; Methionine: T4; Tryptophan + Methionine, T5; Lysine +Tryptophan, T6; Methionine +Lysine, T7; Lysine + Tryptophan +Methionine. Hemoglobin (Hb), white blood cells (WBC), red blood cells (RBC), mean corpuscle volume (MCV), haematocrit (HCT), mean corpuscular hemoglobin (MCH), μL–microliter, fL- femtoliter.

Most of the hematological parameters showed a significant difference (*P*<0.05) between the eight treatments both at the end of the growth experiment and after the bacterial challenge. Hematological parameters exhibited a similar pattern between treatments except that glucose gradually decreased and showed a significant difference (*P*<0.05) among T5, T6 and T7 treatments whereas, T0, T1, and T2 showed insignificant difference (*P*>0.05) (Fig 1). At the end of growth experiment, the hemoglobin (6.91±0.08 g/dl), white blood cells (4.42±0.12 μL), and hematocrit (20.51±0.08%) in theT0 treatment were observed to be the lowest compared to other treatments. All the hematological parameters were significantly (*P*<0.05) higher in the T7 treatment, except for glucose (71.23±1.85 mg/dL), at the end of the growth experiment. The treatments T4, T5, and T6, which were supplemented with synergistic amino acids, showed significantly higher hematological parameters compared to single or non-amino acid supplemented treatments (T1, T2, T3, and T0 treatment).

The glucose level after bacterial challenge was observed to be significantly (*P*<0.05) highest in the +ve T0 treatment. The glucose level of the -ve T0 treatment after the bacterial challenge was observed to be significantly lowest (75.00±0.88 mg/dL), compared to other treatments. All the hematological parameters in the +ve T0 after bacterial challenge were observed to be significantly lowest. The T4, T5, and T6 treatments showed a significantly better response to bacteria compared to the T1, T2, and T3 treatments. All the hematological parameters in T7 treatment were significantly (*P*<0.05) higher after bacterial challenge compared to other treatments (Fig 1).

The blood biochemistry parameters in treatments fed combined amino acids demonstrated a notable increase, exhibiting a statistically significant difference (*P*<0.05) compared to treatments fed with individual and zero amino acids. The levels of triglycerides, ALT, and AST significantly lowered in all treatments compared to +ve T0 (Table 7). At the end of the growth experiment, the neutrophil count (35.21±0.57 μ/L), lymphocytes (65.21±2.61 μ/L), monocytes (3.31±0.05), and eosinophils (2.62±0.05 μ/L) were observed to be significantly lower (P<0.05) in the T0 treatment, compared to the other treatments. The levels of triglycerides (281.00±1.33 mg/dl), ALT (39.21±0.57 unit/L), and AST (30.41±0.57 unit/L) were significantly higher in the T0 treatment compared with other treatments at the end of the growth experiment. The highest levels of lymphocytes (79.02±0.57), monocytes (5.32±0.05), and eosinophils (4.54±0.05) were observed high in T7 treatment at the end of the growth experiment.

**Table 7:** Analysis of biochemical parameters of blood in eight treatments eight treatments at the end of the growth experiment and after the bacterial challenge. Different superscripts across the rows represent the variance between treatments were calculated by Duncan multirange test of one-way ANOVA at *P* < 0.05.

**At the end of the growth experiment**									
**Parameters**	**T0**	**T1**	**T2**	**T3**	**T4**	**T5**	**T6**	**T7**	
Neutrophils (μ/L)	35.21±0.57^a^	35.21±0.57^b^	37.23±0.57^c^	40.02± 0.57^d^	46.00±0.57^f^	45.02±0.57^e^	46.00±0.57^f^	47.00±0.57^g^	
Lymphocytes (μ/L)	65.21±2.61^a^	70.21±0.88^c^	67.21±0.57^b^	72.00±1.51^e^	72.23±0.88^f^	71.21±0.66^d^	75.21±0.57^g^	79.02±0.57^h^	
Monocytes (μ/L)	3.31±0.05^a^	3.32±0.33^b^	3.52±0.33^e^	3.32±0.33^b^	4.81±0.05^d^	5.32±0.05^g^	5.21±0.05^f^	5.32±0.05^g^	
Eosinophils (μ/L)	2.62±0.05^a^	2.82±0.05^e^	3.12±0.57^f^	2.63±0.05^b^	2.72±0.33^d^	2.71±0.05^c^	3.52±0.05^g^	4.54±0.05^h^	
Triglycerides(mg/dl)	281.00±1.33^f^	278.00±0.57^e^	255.03±0.57^d^	199.01±0.57^b^	220.21±0.57^c^	219.21±0.57^c^	189.00±0.57^a^	188.21±0.57^a^	
ALT (Unit/L)	39.21±0.57^g^	38.32±0.57^f^	34.41±0.57^e^	34.41±0.57^e^	30.03±0.5^bd^	29.32±0.57^c^	29.02±0.57^b^	24.03±0.57^a^	
AST (Unit/L)	30.41±0.57^f^	31.03±0.57^g^	30.03±0.57 ^e^	23.04±0.88^d^	21.32±0.66^c^	23.02±0.57^d^	20.32±0.57^b^	18.54±0.57^a^	
Total Protein (g/dl)	6.31±0.15^b^	6.31±0.15^b^	6.41±0.05^c^	6.42±0.05^c^	6.10±0.08^a^	6.31±0.08^b^	6.61±0.05^d^	7.41±0.08^e^	
**At the end of the bacterial challenge**									
**Parameters**	**-ve T0**	**+ve T0**	**T1**	**T2**	**T3**	**T4**	**T5**	**T6**	**T7**
Neutrophils (μ/L)	27.02±0.57^i^	19.10±0.57^a^	21.21±0.57^d^	20.23±0.57^b^	21.00±0.57 ^c^	25.03±0.57^h^	24.21±0.57^g^	23.23±0.57^e^	24.00±0.57^f^
Lymphocytes (μ/L)	68.32±0.33^e^	52.21±1.45^a^	69.01±0.57^f^	66.23±0.57 ^c^	63.23±0.57^b^	67.23±1.22^d^	69.00±0.57^f^	66.42±0.57^c^	69.23±0.88^g^
Monocytes (μ/L)	3.31±0.05^f^	1.63±0.08^a^	1.81±0.05^b^	1.81±0.08 ^b^	1.95±0.03^c^	2.31±0.33^b^	2.11±0.05a^d^	2.11±0.11^d^	2.21±0.05^e^
Eosinophils (μ/L)	2.33±0.33^f^	1.82±0.05^c^	1.62±0.05^b^	1.52±0.08^a^	1.83±0.05^abc^	2.23±0.05^bc^	2.34±0.08^e^	2.21±0.05^d^	2.43±0.05^g^
Triglycerides(mg/dl)	303.00±1.52^a^	385.00±1.85^h^	372.00±1.21^f^	374.02±0.57^g^	323.03±2.84^c^	330.32±0.57^d^	340.00±0.57^e^	338.00±0.88^e^	315.00±2.88^b^
ALT (Unit/L)	37.32±0.57^a^	55.00±0.57^g^	42.21±0.88^e^	44.34±0.57^f^	45.03±0.57^d^	38.34±0.57^b^	40.21±1.21^d^	40.23±1.21^d^	40.04±0.57^c^
AST (Unit/L)	30.05±0.88^a^	47.21±0.88^i^	40.34±1.21^d^	46.34±0.57^h^	45.42±0.88^g^	41.21±0.57^f^	41.02±0.57^e^	35.21±0.57^b^	37.30±1.21^c^
Total Protein (g/dl)	5.42±0.08^h^	3.21±0.03^a^	3.42±0.15^b^	4.92±0.03^e^	4.42±0.25^c^	4.70±0.05^d^	5.41±0.08^d^	5.14±0.11^f^	5.41±0.08^g^

(T0; control, T1; Tryptophan, T2; Lysine; T3; Methionine: T4; Tryptophan + Methionine, T5; Lysine +Tryptophan, T6; Methionine +Lysine, T7; Lysine + Tryptophan +Methionine). alanine aminotransferase (ALT), aspartate aminotransferase (AST), μ/L- micro liter

At the end of the bacterial challenge, the +ve T0 treatment showed the highest levels of triglycerides, ALT, and AST. The total protein level of the -ve T0 treatment after the bacterial challenge was observed to be significantly highest (5.42±0.08 g/dL), compared to other treatments. The triglycerides, ALT, AST in the +ve T0 after the bacterial challenge were also observed to be significantly highest (*P*<0.05). The T4, T5, and T6 treatments exhibited a significantly better response to bacteria compared to the T1, T2, and T3 treatments. Eosinophils, monocytes, lymphocytes, and total protein in the T7 treatment were significantly (*P*<0.05) higher after bacterial challenge compared to other treatments (Table 7).

Catalase (CAT) showed an insignificant (*P*>0.05) difference between T0, and T1, whereas, T2, T3, T4, T5, T6, and T7 treatments showed a significant difference (*P*<0.05). The levels of catalase after the bacterial challenge were significantly different among all treatments. Superoxide dismutase (SOD), and malondialdehyde (MDA) were significantly different (*P*<0.05) among most of the treatment groups after the growth experiment and bacterial challenge (Table 8). The levels of CAT and SOD increased in response to the bacterial challenge in treatment groups. The highest level of CAT (4.58±0.02 μmol/L) and SOD (1.25±0.01μmol/L) were observed in the T7 group. On the other hand, the concentration of MDA gradually decreased in individual or combined amino acids supplemented groups (+ve T0>-ve T0> T1> T2>T3>T4>T5>T6>T7).

**Table 8:** Analysis of catalase (CAT), superoxide dismutase (SOD), and malondialdehyde (MDA) from fish blood serum of eight treatments at the end of the growth experiment and after bacterial challenge. Different superscripts across the rows represent the variance between treatments were applied as a result of one way (Duncan multirange test) at *P* < 0.05.

**At the end of the growth experiment**									
**Parameters**	**T0**	**T1**	**T2**	**T3**	**T4**	**T5**	**T6**	**T7**	
Catalase (μmol/L)	0.25±0.01^a^	0.25±0.01^a^	0.27±0.01^c^	0.26±0.01^b^	0.29±0.01^d^	0.66±0.02^e^	1.36±0.01^f^	2.78±0.03^g^	
SOD (μmol/L)	0.13±0.01^a^	0.14±0.01^b^	0.18±0.01^d^	0.17±0.01^c^	0.26±0.01^e^	0.27±0.01^f^	0.38±0.02^g^	1.18±0.01^h^	
MDA (μmol/L)	1.25±0.02^g^	0.25±0.01^e^	0.72±0.23^f^	0.23±0.01^d^	0.22±0.01^c^	0.15±0.01^a^	0.17±0.01^b^	0.14±0.01^a^	
**At the end of the bacterial challenge**									
**Parameters**	**-ve T0**	**+ve T0**	**T1**	**T2**	**T3**	**T4**	**T5**	**T6**	**T7**
Catalase (μmol/L)	0.58±0.01^b^	0.15±0.01^a^	1.25±0.01^c^	2.11±0.01^d^	2.13±0.01^d^	3.23±0.01^g^	2.30±0.01^e^	2.54±0.01^f^	4.58±0.02^h^
SOD (μmol/L)	0.14±0.01^a^	0.19±0.01^b^	0.33±0.01^d^	0.31±0.01^c^	0.15±0.02^a^	0.35±0.02^e^	0.43±0.01^f^	0.45±0.01^g^	1.25±0.01^h^
MDA (μmol/L)	0.14±0.01^a^	1.54±0.01^e^	0.32±0.01^d^	0.25±0.02^c^	0.33±0.01^d^	0.24±0.01^b^	0.23±0.02^b^	0.24±0.01^b^	0.14±0.01^a^

(T0; control, T1; Tryptophan, T2; Lysine; T3; Methionine: T4; Tryptophan + Methionine, T5; Lysine +Tryptophan, T6; Methionine +Lysine, T7; Lysine + Tryptophan +Methionine), μmol/L: micro moles per liter.

**Fig 2:**
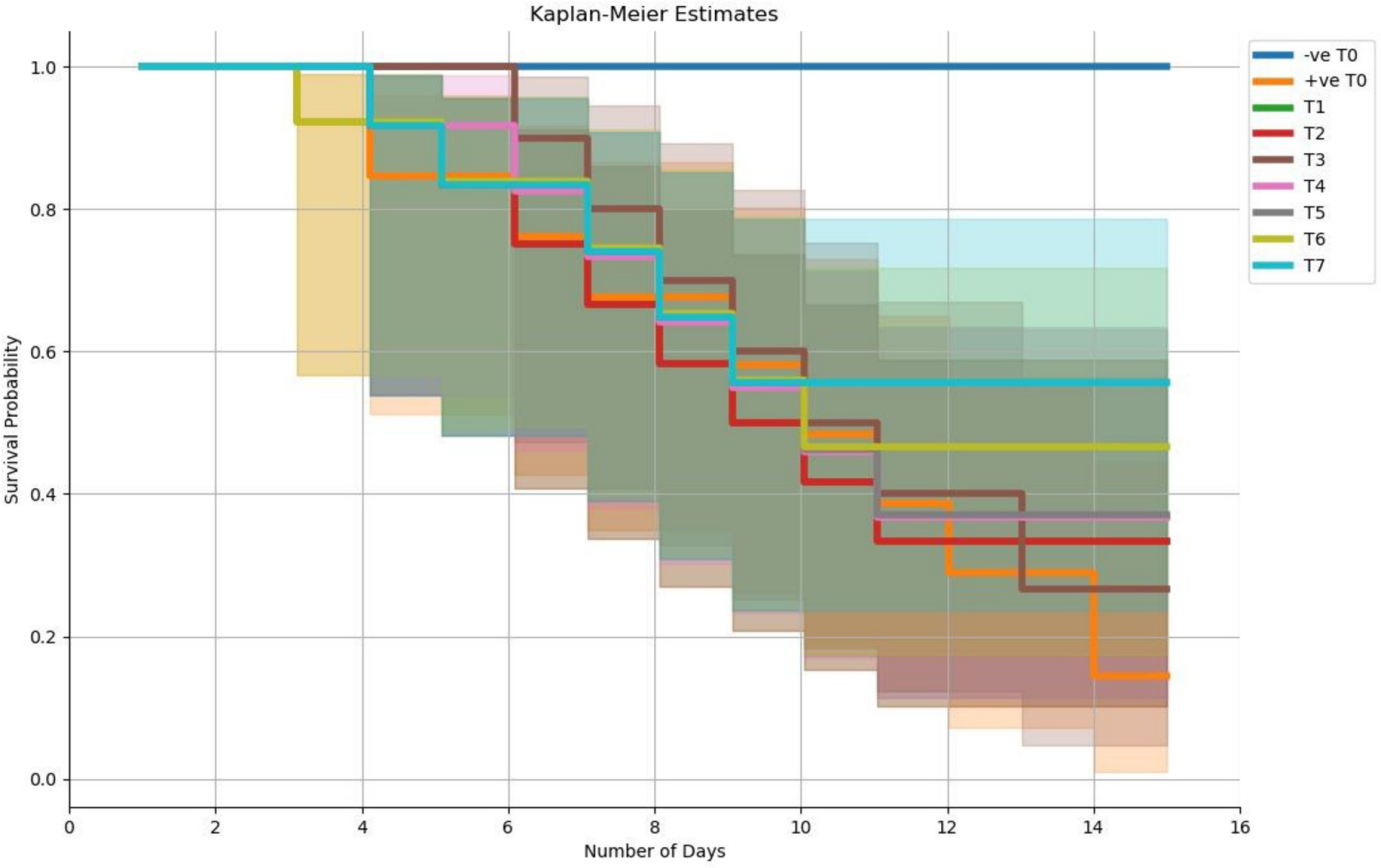
Kaplan-Meier survival curves of striped catfish following infection by immersion challenge with *S*. ***aureus*.** The results correspond to the survival percentage during 15 days post-infection of three replicates (n = 15/treatment). Kaplan-Meier survival data was analyzed by OriginPro software.

Survival rate after the bacterial challenge was presented through the Kaplan Meier survival curve. The best survival rate was observed in the T7 treatment compared to other treatments. The +ve T0 treatment showed less survival rate and high mortality (Fig 2).

### 3.5 Histological analysis

**Fig 3:**
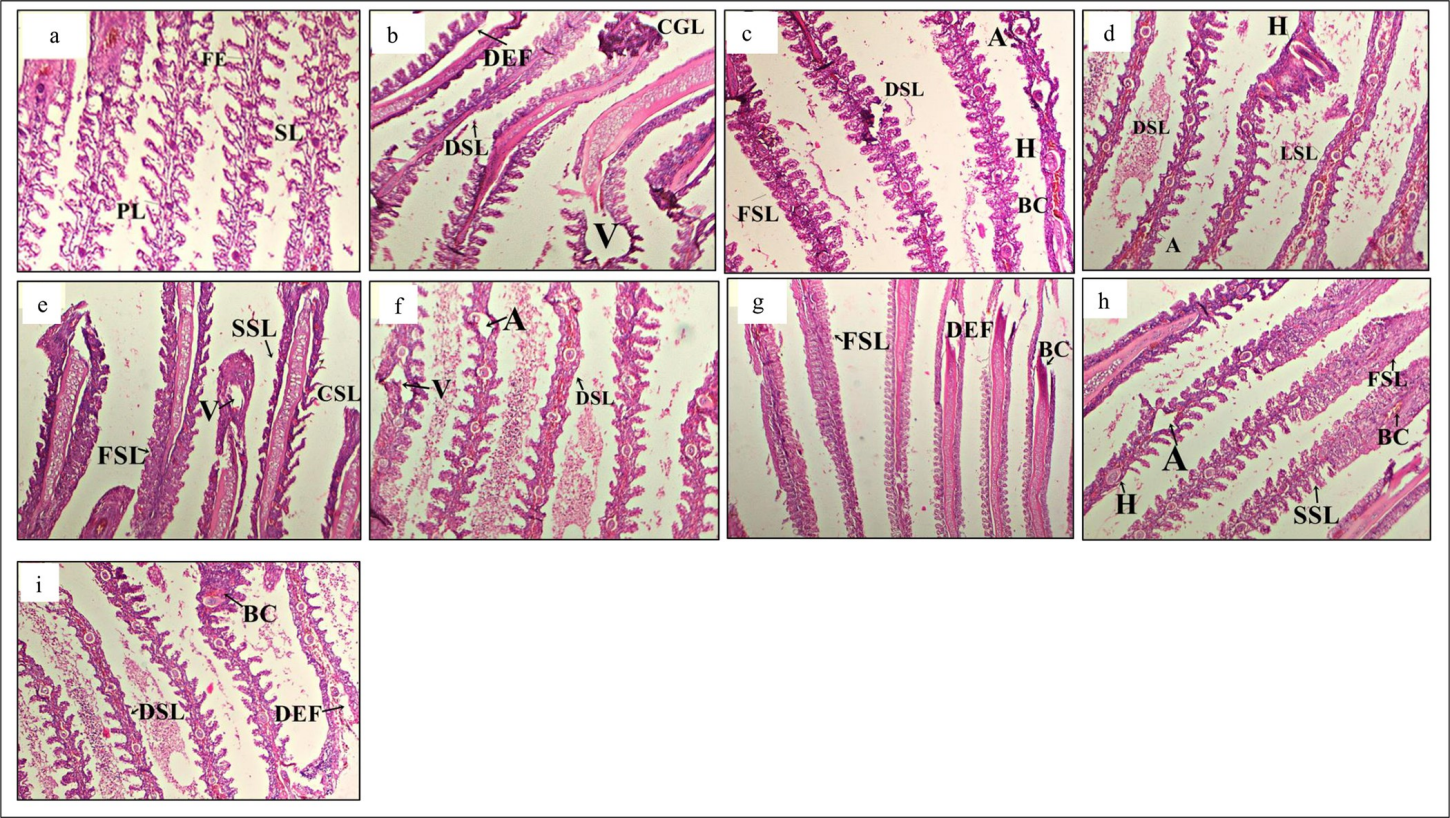
Histological changes in gills. Light micrographs of a paraffin section stained with eosin (40x). a; gills in -ve T0, b; gills t in +ve T0, c; gills T1, d; gills in T2, e; gills in T3, f; gills in T4, g; gills in T5, h; gills in T6, I; gills in T7. Primary lamellae, SL; Secondary lamellae, FSL; Fusion of secondary lamellae, DSL; Degeneration of secondary lamellae, HT; Hypertrophy, DPL; Degeneration of primary lamellae, TD; Tissue debris, A; Aneurism, BC; Blood congestion, V; vacuolation.

**Fig 4:**
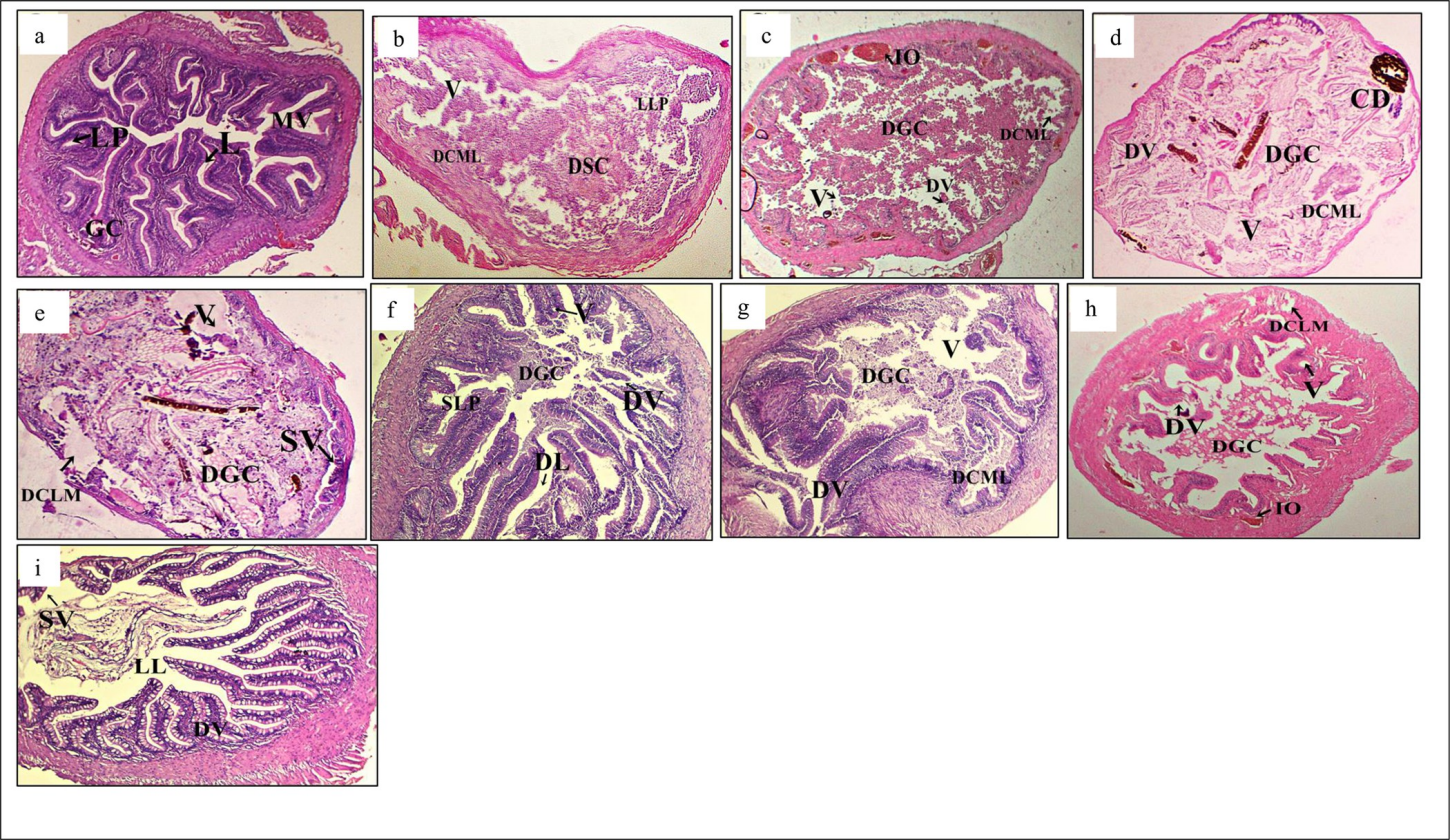
Histological changes in gut. Light micrographs of a paraffin section stained with eosin (40x). a; gut in -ve T0, b; gut in +ve T0, c; gut in T1, d; gut in T2, e; gut in T3, f; gut in T4, g; gut in T5, h; gut in T6, I; gut in T7. GC; Goblet cells, LP; Laminar propria, N; Nucleus, L; Lumen, CE; Columnar epithelium, FV; Fusion of villi, LL; Large lumen, FLV; Flattened villi, DCML; Damaged circular muscle layer, DL; Distended lumen, DLML; Damaged longitudinal muscle layer, VF; Vacuole formation, SLP; Swelling of lamina propria, CCA; Cracked clay appearance of the tissues, SLML; Swelling of longitudinal muscle layer, DGC; Damaged goblet cells, DMM; Disarrangement of muscularis mucosa.

Several histopathological alterations were observed in the gill structure in all treatments (Fig 3A–3I). The histology of gills in the -ve T0 group exhibited the typical epithelial cell lining of lamellae (Fig 3A). In contrast, the groups exposed to *S*. *aureus* showed various structural changes, such as hemorrhage, intracellular oedema, disruption of gills with notable hypertrophy, loss of horizontal shaft with mucous membrane cellular proliferation (Fig 3B–3I). The +ve T0 treatment (Fig 3B) showed the highest anomalies and mortality. The gut structure of different treatments showed several pathologies (Fig 4A–4I). Histopathological analysis of the -ve T0 treatment showed a normal or less alterations in goblet cells, villi, and nucleus (Fig 4A). Meanwhile, the other treatments revealed structural anomalies such as excessive hypertrophy, the villi tended to fuse (FV), and the mucosal lining sloughed off, eventually leading to the large lumen (LL) (Fig 4B–4I).

**Fig 5:**
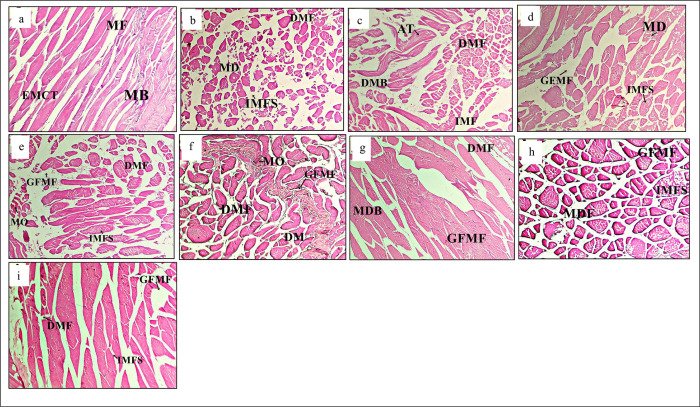


**Fig 6:**
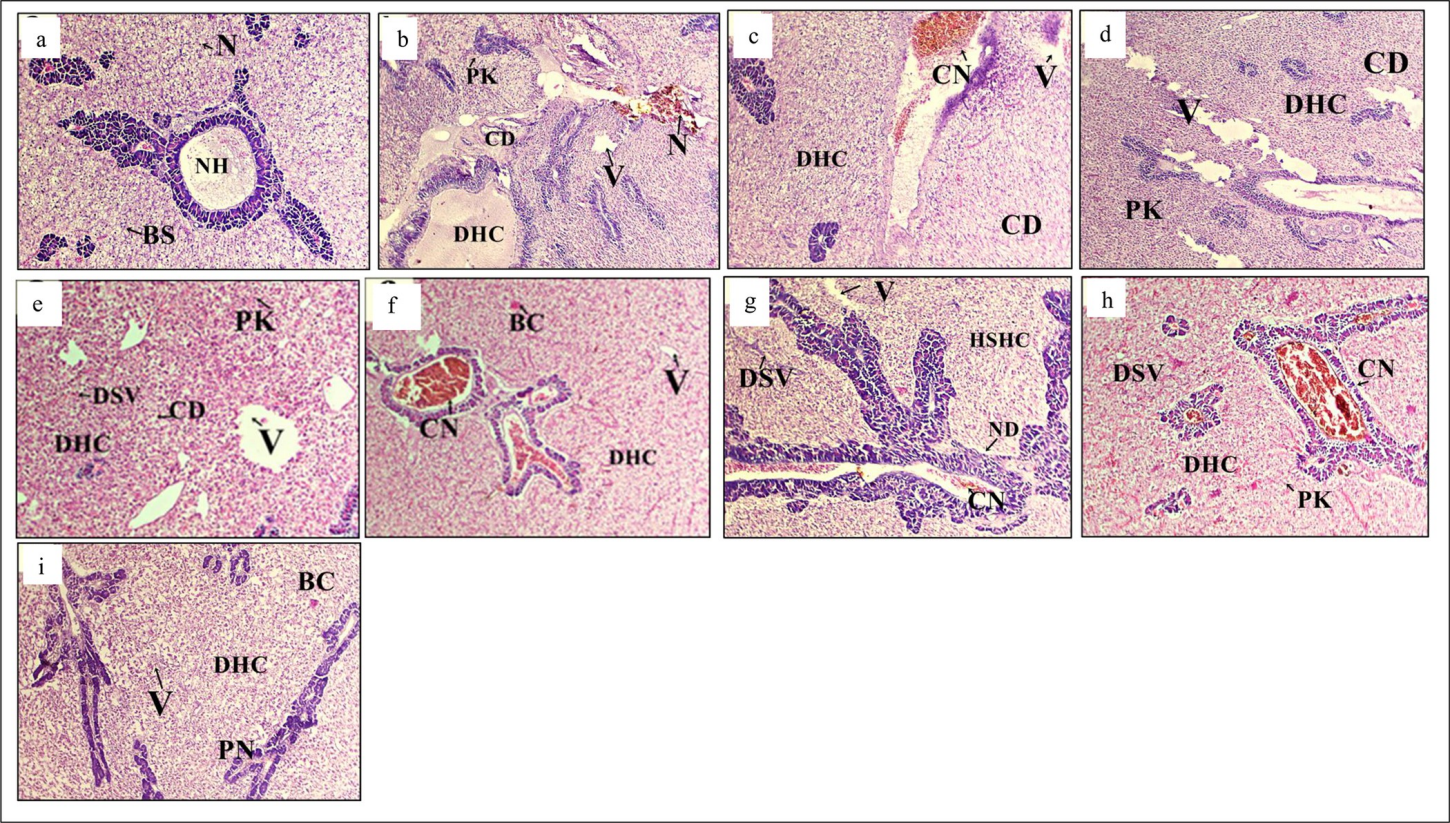
Histological changes in liver. Light micrographs of a paraffin section stained with eosin (40x). a; liver in -ve T0, b; liver in +ve T0, c; liver in T1, d; liver in T2, e; liver in T3, f; liver in T4, g; liver in T5, h; liver in T6, I; liver in T7. NH; Normal hepatocytes, GC; Granular cytoplasm, BC; Blood congestion HD; Hepatocyte generation, CSN; Central spheroidal hepatocyte nucleus, N; Cell necrosis, PN; Pyknotic nuclei, CD; Cytoplasmic degeneration, IEF; Infiltration of oedematous fluid, rCV; Rupturing of the central vein, V; Vacuolization of hepatocytes. DHC; degeneration of hepatocytes.

Several anomalies were observed in muscles structures of the eight treatments after the bacterial challenge (Fig 5A–5I). Muscle structures of the -ve T0 treatment showed less or no abnormalities (Fig 5A) as compared to other treatments. Whereas, different treatments showed structural changes like, muscle fibers degeneration, vacuole destabilization in muscle bundles and the increased inter myofibrillar space (IMFS) (Fig 5B–5I). The highest pathological alterations were observed in the muscles of +ve T0 treatment (Fig 5B). Liver in different treatments showed significant abnormalities (Fig 6A–6I). Treatment bathed with phosphate buffer saline and fed with zero amino acid supplement (-ve T0) showed normal hepatocytes, endothelium, and serous membrane that contained blood vessels (Fig 6A). On the other hand, different treatments showed pathologies such as necrosis, multinucleated nucleolus, oedema, hemosiderin, hematoma, intravenous tissue necrosis, edematous fluid intrusions (Fig 6B–6I).

**Fig 7:**
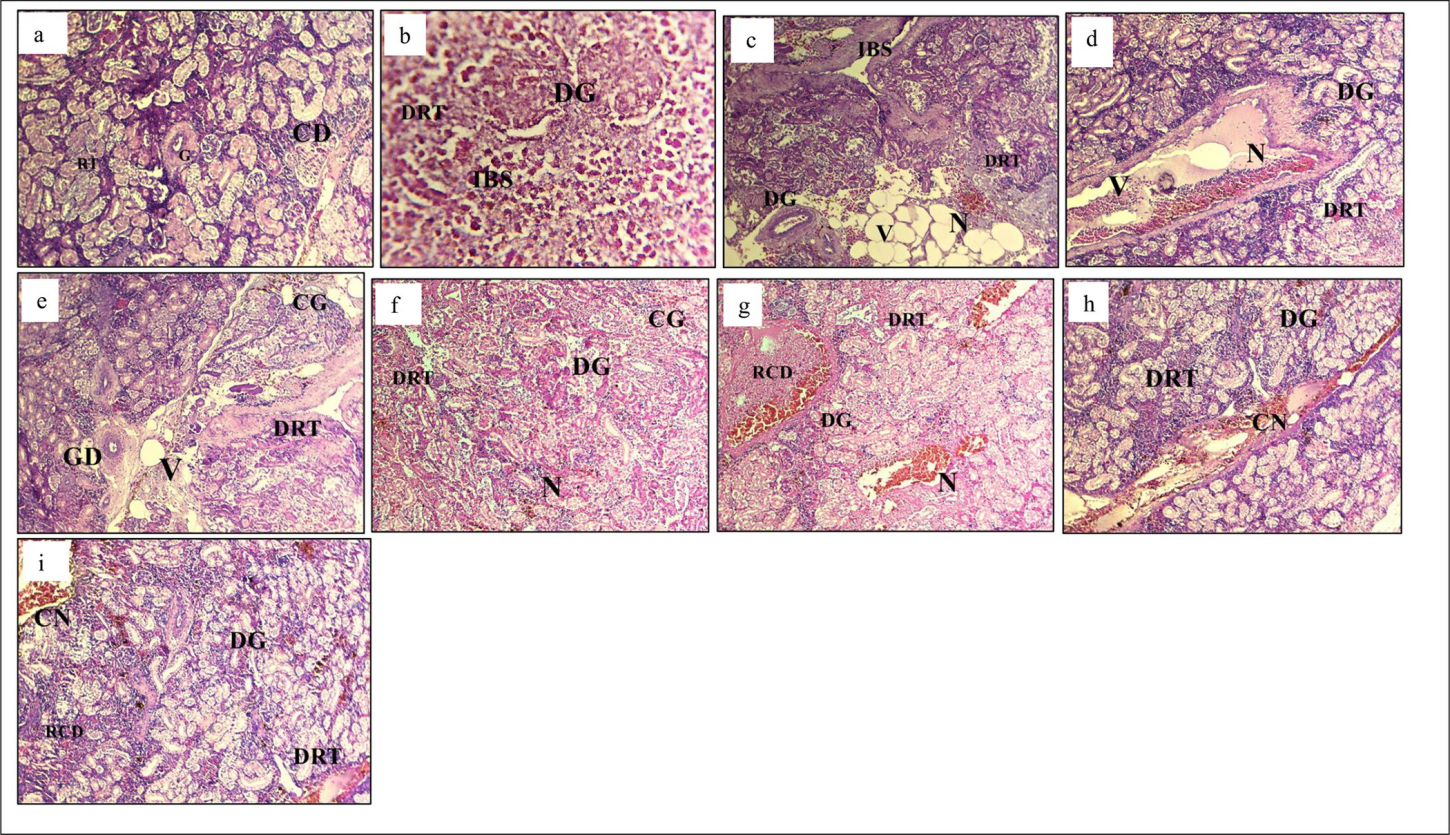
Histological changes in kidney. Light micrographs of a paraffin section stained with eosin (40x). a; kidney in -ve T0, b; kidney in +ve T0, c; kidney in T1, d; kidney in T2, e; kidney in T3, f; kidney in T4, g; kidney in T5, h; kidney in T6, I; kidney in T7. G; Glomerulus, CD; Collecting duct, DG; Degenerative glomerulus, IBS; Increased bowman space, FRT; Fusion of renal tubule, DRT; Degenerative renal tubule, CG; Congestion of glomerulus, N; Necrosis, H; Hemorrhage, A; Atrophy.

Kidney structure of eight treatments exhibited anomalies. Less or no structural abnormalities were observed in kidney structure of -ve T0 treatment (Fig 7A). However, the +ve T0 treatment displayed the highest structural abnormalities among all other treatments (Fig 7B). Severe structural changes among treatments were observed (T1>T2>T3>T4>T5>T6>T7) (Fig 7C–7I).

## 4. Discussion

The present study showed that striped catfish fed a diet without methionine, lysine, and tryptophan supplementation exhibited poor growth. Total body weight, hepatosomatic index, and specific growth rate were lower in the T0 treatment. However, these parameters gradually increased in dietary treatments with combined amino acids as compared to individual amino acid supplementation. The highest growth rate probably accounts for better feed utilization efficiency, a faster absorption rate as well as greater availability of free methionine, lysine, and tryptophan in tissues to be utilized [62]. Present findings were similar to the experimental results in Chinese sucker (*Myxocyprinus asiaticus*) [63], yellowtail (*Seriola quinqueradiata*) [64], common carp [65], large yellow croaker (*Pseudosciaena crocea*) [66], rohu [67], Atlantic salmon (*Salmo salar*) [68], juvenile hybrid striped bass (*Morone saxatilis*) [69], and juvenile Jian carp (*Cyprinus carpio*) [70].

Fish growth is positively associated with the accretion of protein, fat, and other nutrients [5]. The present study demonstrates a significant decrease (*P*<0.05) in crude lipid, and an increase in crude protein in most treatments compared with those fed with the T0 diet. The highest protein level was observed in fish fed with the T7 diet (22.75±0.01%), which may be due to high activation of the target of rapamycin (TOR) signaling pathway. The TOR signaling pathway is involved in improving protein synthesis and growth in fish [71]. These results coincide with other fish species, including silver perch [72], yellow crocker [73], and silver pompano [74]. The growth of fish is also intricately linked to their digestive and absorptive capabilities, which, in turn, depends upon the performance of digestive and brush border enzymes [75]. At the end of the present study, the activities of intestinal amylase, trypsin, and lipase exhibited a decline in fish fed a diet without methionine supplementation (T0), while they displayed an increase in fish that were fed diets containing optimal levels of methionine, lysine, and tryptophan.

In the present research, the comprehensive hematological profile following the growth experiment and the bacterial challenge underwent significant modification as a result of either tryptophan, or methionine or lysine supplementation. Furthermore, a notably higher number of white blood cells (WBC) was noted in the all treatments compared with T0. This augmentation in WBC count aligns with previous findings in European seabass [76], where the provision of varying levels of methionine hydroxy analogue led to increased survival rates and enhanced humoral and cellular responses after injection of *Aeromonas hydrophila* [77]. Tryptophan and methionine have been substantiated to play pivotal roles in bolstering the immune response, while concurrently demonstrating the ability to modulate metabolic pathways implicated in enhancing the efficacy of the immune system [78].

In fish, intestinal health has been correlated with the intestinal physical barrier, which mainly consists of intestinal epithelial cells and tight junction proteins (such as claudins, occludin and ZO-1) [79]. Intestinal inflammation is accompanied by the excessive production of reactive oxygen species (ROS), which causes lipid peroxidation and protein oxidation damage in intestinal epithelial cells [70]. Lipid peroxidation and protein oxidation in tissues can be reflected by the MDA [80]. In this study, dietary supplementation of amino acids, either individual or combined, showed a significant decrease in the serum MDA contents of striped catfish, suggesting that an appropriate amino acid level might have inhibited lipid peroxidation and protein oxidation in the fish intestine [81]. The possible inhibition of oxidative damage is considered closely related to the improvement in non-enzymatic and enzymatic antioxidant capacities in fish [82]. Previous studies indicated that nutrients, such as histidine [83, 84], and lysine, increased the glutathione (GSH) content in the intestine of fish. Additionally, SOD and GPx are major antioxidant enzymes in fish [85]. In present study, the activities of SOD and CAT in the serum were the highest in the appropriately supplemented diets, suggesting that amino acids could improve the enzymatic antioxidant capacity in fish.

The histological alterations during the bacterial challenge test correlated with haemato-biochemical and antioxidant enzyme data [86]. This study elucidates notable variations in various tissues, including muscles, gills, kidneys, liver, and gut. The greatest tissue damage was observed in the +ve T0 treatment. The gills are particularly susceptible to waterborne pathogens due to their perpetual exposure to the external environment [87, 88]. In +ve T0 treatment, the gills displayed a significant prevalence of histological abnormalities when compared to other treatments. This result in erythrocytes congestion within the marginal channel [89]. In contrast, the liver histology of amino acid treated groups showed characteristics reminiscent of those found in negative control group (-ve T0). The liver’s impaired ability to efficiently remove foreign particles results in the degeneration of hepatocytes and congestion within sinusoid’s [90]. The presence of extracellular toxin generated by *S*. *aureus* might be the underlying factor responsible for the formation of lipid vacuoles and the occurrence of necrosis in the liver. Comparable hepatic irregularities, including the infiltration of lymphocytes, focal necrosis and the presence of cytoplasmic fat vacuoles, have been similarly observed in carp [91].

Fish exposed to *S*. *aureus*, kidney tissues displayed severe necrosis and observable changes in the glomeruli. Notably, the glomerular epithelium in the kidney of catfish afflicted by *S*. *aureus* exhibited noticeable histological alterations [92]. A pronounced elevation in the height of intestinal villi and reduction in adverse effects of *S*. *aureus* within the dietary groups might be due to action of amino acids inhabiting the intestine, causing consequent reduction in pH and inhibiting fermenting indigestible carbohydrates. Histopathological results support and confirm our examined hematological parameters and consistent with previous findings of pathological examination of *S*. *aureus*.

## 5. Conclusion

The present results demonstrated that dietary supplementation of methionine, lysine, and tryptophan in striped catfish significantly improved growth performance and enhanced digestive and absorptive function. Additionally, amino acid supplementation provided protection to the hepatopancreas and intestine from lipid peroxidation and protein oxidation by enhancing enzymatic antioxidant capacity (SOD, Catalase and MDA activities). Based on observed weight gain, intestinal trypsin and hepatopancreatic anti-hydroxy radical activities, the dietary requirement for methionine, lysine, and tryptophan in catfish is suggested to be 5g/kg. The most favorable outcomes in striped catfish were observed in fish supplemented with the combined three amino acids in the diet (T7). Further studies are needed to elucidate the specific molecular mechanisms through which these amino acids mediate antioxidant defense in fish.

## Supporting information

S1 Data (XLSX)
